# Y-box binding protein-1 promotes hepatocellular carcinoma-initiating cell progression and tumorigenesis via Wnt/β-catenin pathway

**DOI:** 10.18632/oncotarget.13733

**Published:** 2016-12-01

**Authors:** Hsiao-Mei Chao, Hong-Xuan Huang, Po-Hsiang Chang, Kuo-Chang Tseng, Atsushi Miyajima, Edward Chern

**Affiliations:** ^1^ niChe Laboratory for Stem Cell and Regenerative Medicine, Department of Biochemical Science and Technology, National Taiwan University, Taipei, Taiwan; ^2^ Department of Pathology, Wan Fang Hospital, Taipei Medical University, Taipei, Taiwan; ^3^ Institute of Molecular and Cellular Biosciences, The University of Tokyo, Tokyo, Japan

**Keywords:** Y-box binding protein 1, tumor-initiating cell, HCC, Wnt, stemness

## Abstract

Y-box binding protein-1 (YB-1) is a pleiotropic molecule that binds DNA to regulate genes on a transcriptional level in the nucleus and binds RNA to modulate gene translation in the cytoplasm. In our previous studies, YB-1 was also characterized as a fetal hepatic protein that regulates the maturation of hepatocytes and is upregulated during liver regeneration. Moreover, YB-1 has been shown to be expressed in human hepatocellular carcinoma (HCC). However, the role of YB-1 in HCC remains unclear. Here, we aimed to characterize the role of YB-1 in HCC. Based on the results of loss-of-function in HCC and gain-of-function in mice liver using hydrodynamic gene delivery, YB-1 promoted hepatic cells proliferation *in vitro* and *in vivo*. YB-1 was also involved in HCC cell proliferation, migration, and drug-resistance. The results of extreme limiting dilution sphere forming analysis and cancer initiating cell marker analysis were also shown that YB-1 maintained HCC initiating cells population. YB-1 also induced the epithelial-mesenchymal transition and stemness-related gene expression. Knockdown of YB-1 suppressed the expression of Wnt ligands and β-catenin, impaired Wnt/β-catenin signaling pathway and reduced the numbers of HCC initiating cells. Moreover, YB-1 displayed nuclear localization particularly in the HCC initiating cells, the EpCAM+ cells or sphere cells. Our findings suggested that YB-1 was a key factor in HCC tumorigenesis and maintained the HCC initiating cell population.

## INTRODUCTION

Y-box binding protein 1 (YB-1) belongs to the family of cold shock binding proteins and binds to the nucleotide sequence invert CCAAT to regulate gene expression. The intracellular distribution of YB-1 determines its functions. YB-1 binds DNA in the nucleus and RNA in the cytoplasm to modulate the transcription and translation of genes, respectively. YB-1 is upregulated in some cancers, such as breast, prostate, and ovarian cancers, and functions as a proto-oncogene [1–3]. Previous studies have shown that YB-1 promotes the expression of multidrug resistance genes, thereby enhancing drug resistance in tumors [4]. YB-1 has also been reported to increase the expression of genes encoding cyclin A and cyclin B, which are associated with cell cycle progression [5]. In breast cancer cells, YB-1 promotes the transcription of CD44 and CD49f [6]. Moreover, YB-1 binds to mRNAs encoding proteins involved in the epithelial-mesenchymal transition (EMT) to regulate gene translation and promote the EMT [7]. Studies have shown that microRNAs (miRNAs) or small molecules target YB-1 to inhibit metastasis of osteosarcoma and prostate cancer [8, 9]. Taken together, these data imply that YB-1 may play a critical role in the tumorigenesis and progression of cancers.

In our previous study, YB-1 was shown to be highly expressed in E12 mouse fetal liver and then exhibit decreased expression with maturity [10]. The expression of YB-1 increases again during liver injury and regeneration. During liver development, YB-1 regulates the transcription of C/EBPα and modulates the expression of carbamoyl phosphate synthetase-1 (CPS-1), playing a role in ammonia metabolism in hepatocytes [10]. Moreover, in the normal adult liver, YB-1 is not expressed or is maintained at a very low level [10, 11]; however, in the fetal and regenerated liver, YB-1 is upregulated. Thus, the expression of YB-1 may be associated with the immaturity and proliferation of hepatocytes.

Hepatocellular carcinoma (HCC) is the most common liver cancer worldwide and is associated with a high mortality rate. The biological function of YB-1 in HCC is still unclear. In 2005, Yasen et al demonstrated that YB-1 was expressed in human HCC [11], with 89% of patients showing positivity for YB-1 in HCC cells. Moreover, YB-1 is typically localized in the cytoplasm; however, HCC cells from some patients showed both intranuclear and cytoplasmic localization; this differential expression pattern was found to be associated with a poor prognosis and low survival rate. Despite these studies, the role of the cellular localization of YB-1 in HCC is poorly understood [11]. Increasing evidence has shown that some stem cell-like cancer cells with self-renewal and heterogeneous properties can act as tumor-initiating cells during tumorigenesis, including HCC. Such stem cell-like cancer initiating cells show higher chemoresistance/radioresistance and higher potential to metastasize [12]. Moreover, some surface markers of fetal hepatoblasts or hepatic progenitor cells, such as EpCAM, CD90, and CD133, can also act as surface markers of HCC initiating cells [13–15]. YB-1 plays an essential role in fetal hepatoblasts and during liver regeneration; thus, YB-1 may participate in HCC tumorigenesis and could be associated with the characteristics of cancer initiating cells.

Accordingly, in the present study, we investigated the significance of YB-1 in HCC and HCC stem cell-like cells to clarify the role of this protein in HCC.

## RESULTS

### YB-1 promoted HCC cell proliferation and colony formation

**Figure 1:**
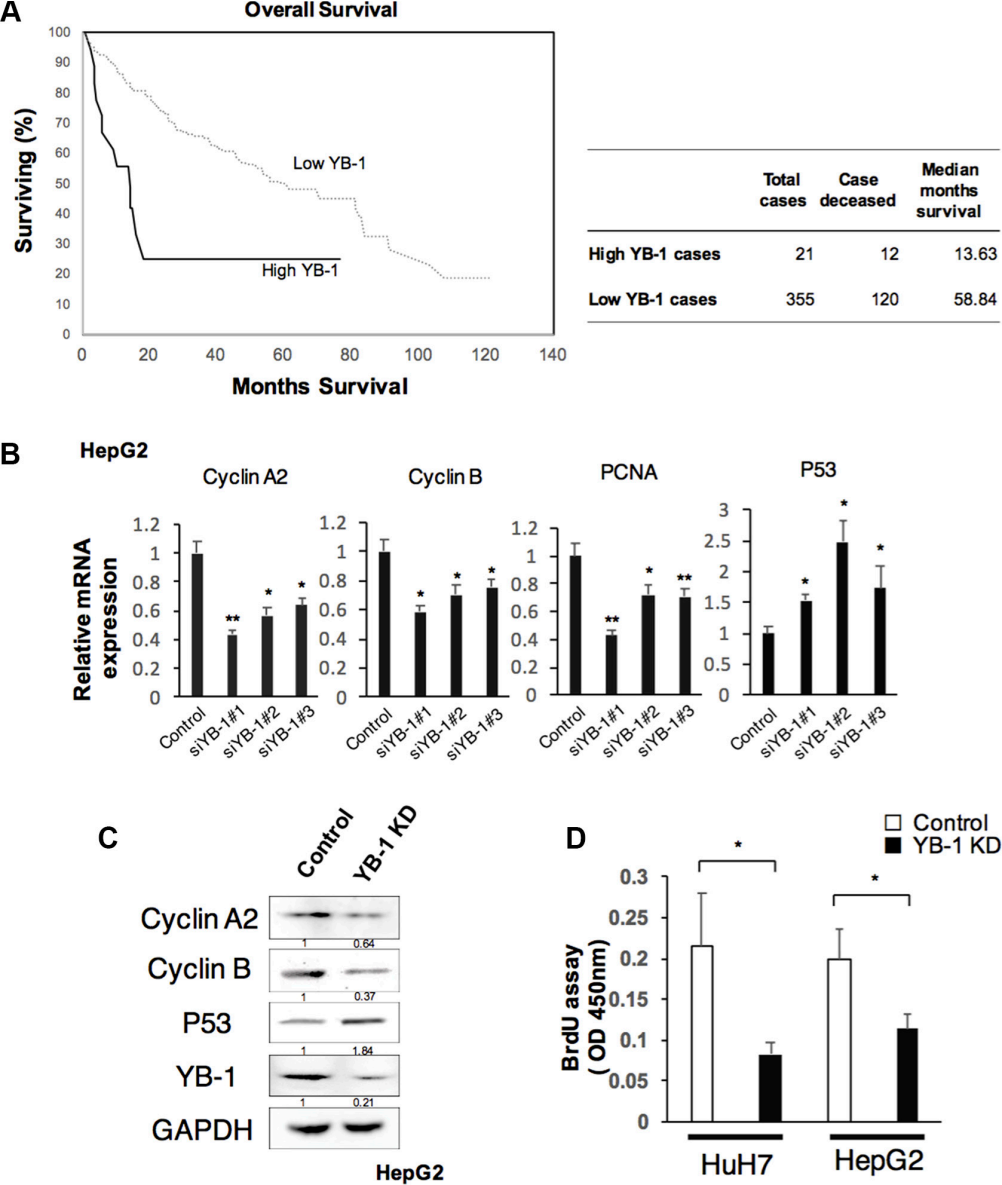
YB-1 promoted HCC proliferation (**A**) Patients with high YB-1 expressed HCC were associated with poor survival. Kaplan-Meier curves of overall survival of patients with hepatocellular carcinoma in cBioportal with TCGA dataset stratified by YB-1 expression. According to cBioportal, High YB-1 indicated YB-1 gene amplification or mRNA upregulation. Low YB-1 indicated no gene alteration of YB-1 in the tumor cases of TCGA sets. (**B**) Knock-down YB-1 in HepG2 cells decreased the expression of proliferation-related genes, Cyclin A2, Cyclin B, and PCNA, and up-regulated tumor suppressor gene P53. The hepatoma cell line, HepG2 was transfected with YB-1 siRNA. Relative expression of Cyclin A2, Cyclin B, PCNA and P53 in HepG2 were analyzed by real-time PCR. Expression levels were normalized to that of GAPDH. Each bar represents the means of three determinations ± SD. *p < 0.05 and **p < 0.01 among the indicated groups compared with control group. (**C**) The protein expressions of Cyclin A2, Cyclin B, and P53 in HepG2 were analyzed by western blot. The protein expression was normalized to GAPDH. (**D**) Knockdown of YB-1 inhibited cell proliferation rate in HCC cells. The proliferation rate of HCC cells was measured by BrdU assay. Each bar represents the means of three determinations ± SD. *p < 0.05 among the indicated groups.

YB-1 has been reported to be expressed in human HCC. We investigated the amplification status of YB-1 in the TCGA dataset using cBioPortal, and the importance of YB-1 overexpression on survival of HCC patients [16, 17]. Kaplan–Meier analysis of patients dichotomized on median YB-1 expression demonstrates poorer overall survival for patients with high versus low YB-1 expressing tumors in the TCGA dataset (Figure 1A). In order to elucidate the function of YB-1 in HCC, we first examined the expression of YB-1 in different HCC cell lines. YB-1 was expressed in HCC cell lines (Supplementary Figure S1).

During fetal liver development and liver regeneration in mice, YB-1 upregulates cyclin A and cyclin B to modulate cell proliferation [10]. To examine whether YB-1 was involved in HCC proliferation, we knocked down YB-1 in HCC cells and measured the expression of proliferation related genes and proliferative ability of HCC cells. Genes encoding cyclin A, cyclin B, and proliferating cell nuclear antigen (PCNA), which are all related to proliferation, were downregulated in YB-1-knockdown cell lines; however, the gene encoding p53 was upregulated (Figure 1B and 1C). YB-1-knockdown cells also reduced the proliferative ability by BrdU assay (Figure 1D). These results showed that HCC cell proliferative activity was decreased in YB-1-knockdown cell lines.

**Figure 2:**
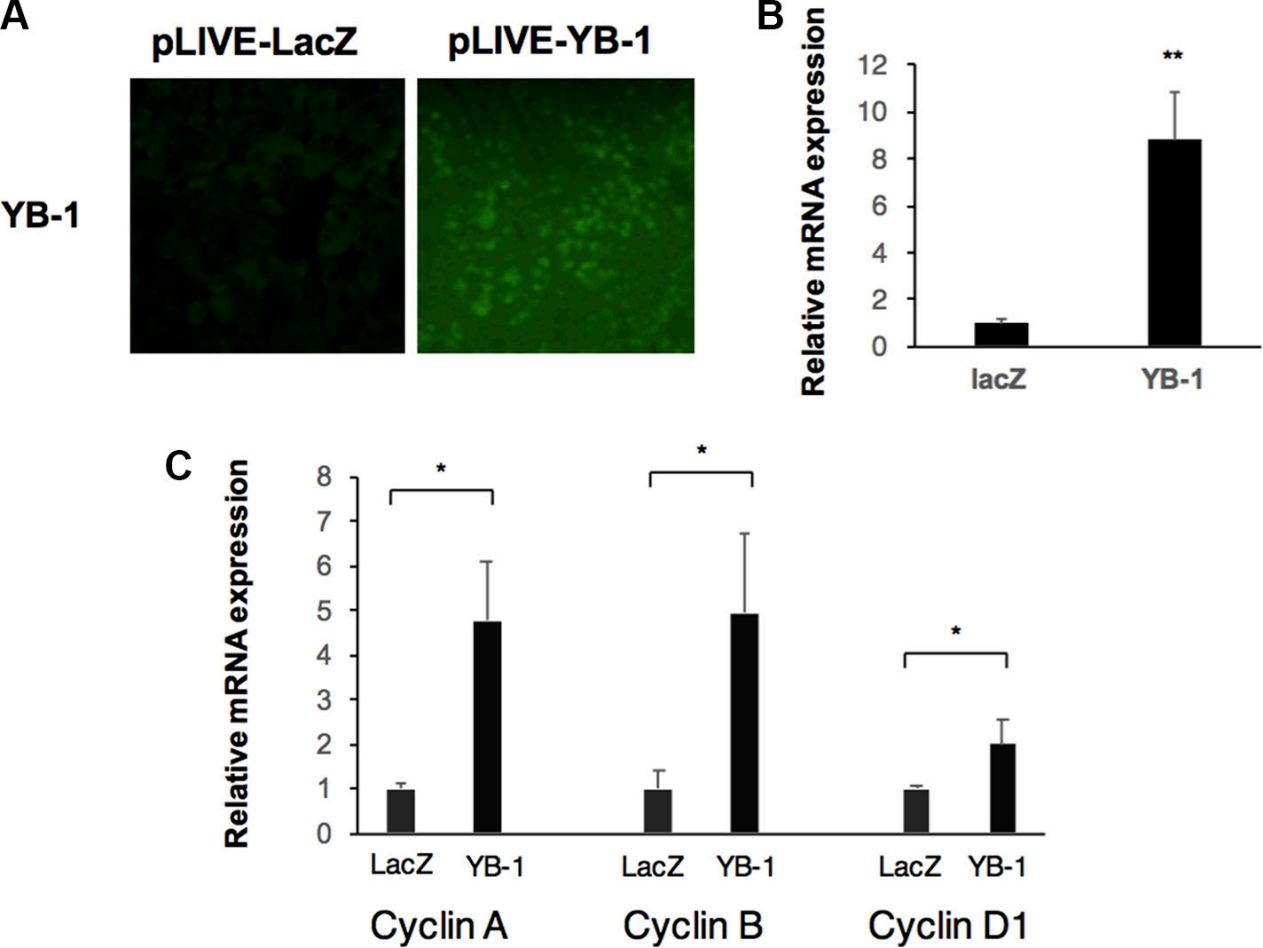
YB-1 induced proliferation genes in mice (**A**) Overexpression of YB-1 in hepatocytes of mice by hydrodynamic gene delivery. Hepatocyte specific expression vector (pLIVE-YB-1) and the control vector (pLIVE-LacZ) were force-expressed in the liver of 6 weeks old mice by hydrodynamic gene delivery method. After 48 hours, the mice livers were stained with YB-1 antibody. (**B**, **C**) Proliferation genes were upregulated in YB-1 overexpressed liver. Relative expression of YB-1and cell cycle related genes in mice liver (*n* = 3) were analyzed by real-time PCR. Expression levels were normalized to that of GAPDH. Each bar represents the means of three determinations ± SD. *p < 0.05 and **p < 0.01 among the indicated groups.

It is difficult to determine the functions of YB-1 in HCC cell lines by gain-of-function mutations owing to the expression of YB-1 in HCC cells. However, YB-1 is not expressed or is expressed at very low levels in adult hepatocytes, and hydrodynamic gene delivery is an efficient method for transiently overexpressing YB-1 in adult liver cells. Compared with the control mouse liver, livers showing overexpression of YB-1 exhibited increased cyclin D, cyclin A, and cyclin B expression at 48 h after gene delivery (Figure 2).

**Figure 3:**
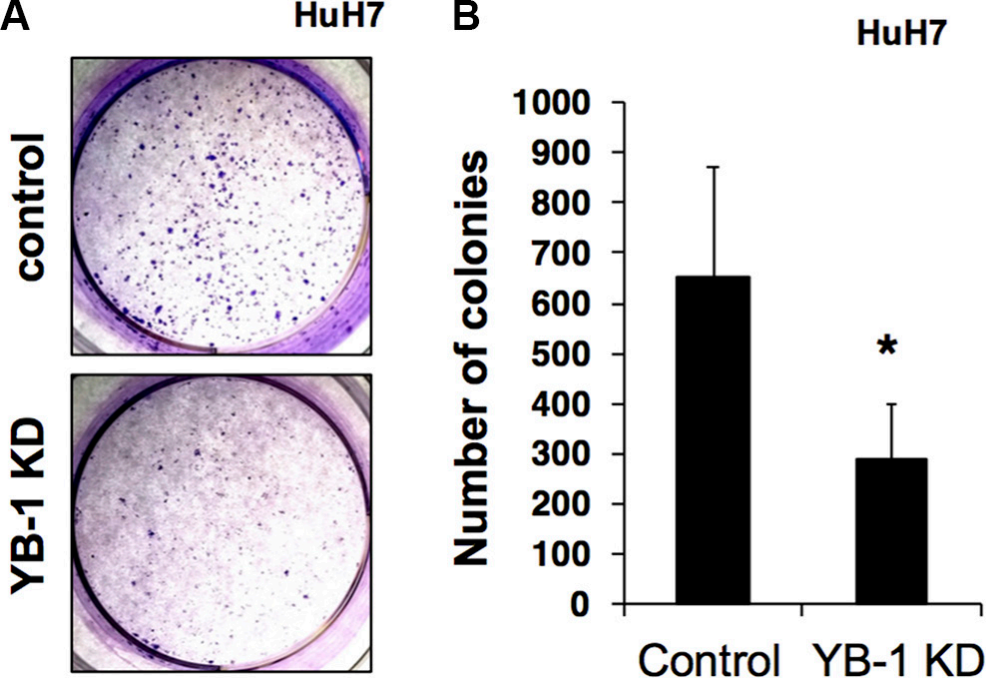
YB-1 KD HCC cells reduced colony formation ability (**A**) The control and YB-1 KD clones of HuH7 cells were seeded at low density in individual wells of a standard 6-well plate and grew for 14 days in 3% FBS DMEM. Colonies were visualized by crystal violet staining (A) and the numbers of colonies were counted (**B**). The ability of colony formation was significantly lower in the YB-1 KD cells group compared with control cells. Each bar represents the means of three determinations ± SD. *p < 0.05 among the indicated groups.

Next, colony formation assays were carried out to investigate the long-term effects of YB-1 on the proliferation and tumorigenesis of hepatoma cells. As shown in Figure 3, the colony-forming ability of YB-1-knockdown cells was reduced. Thus, these results suggested that YB-1 increased the proliferative activity of hepatoma cells.

### YB-1 function was associated with HCC migration

**Figure 4:**
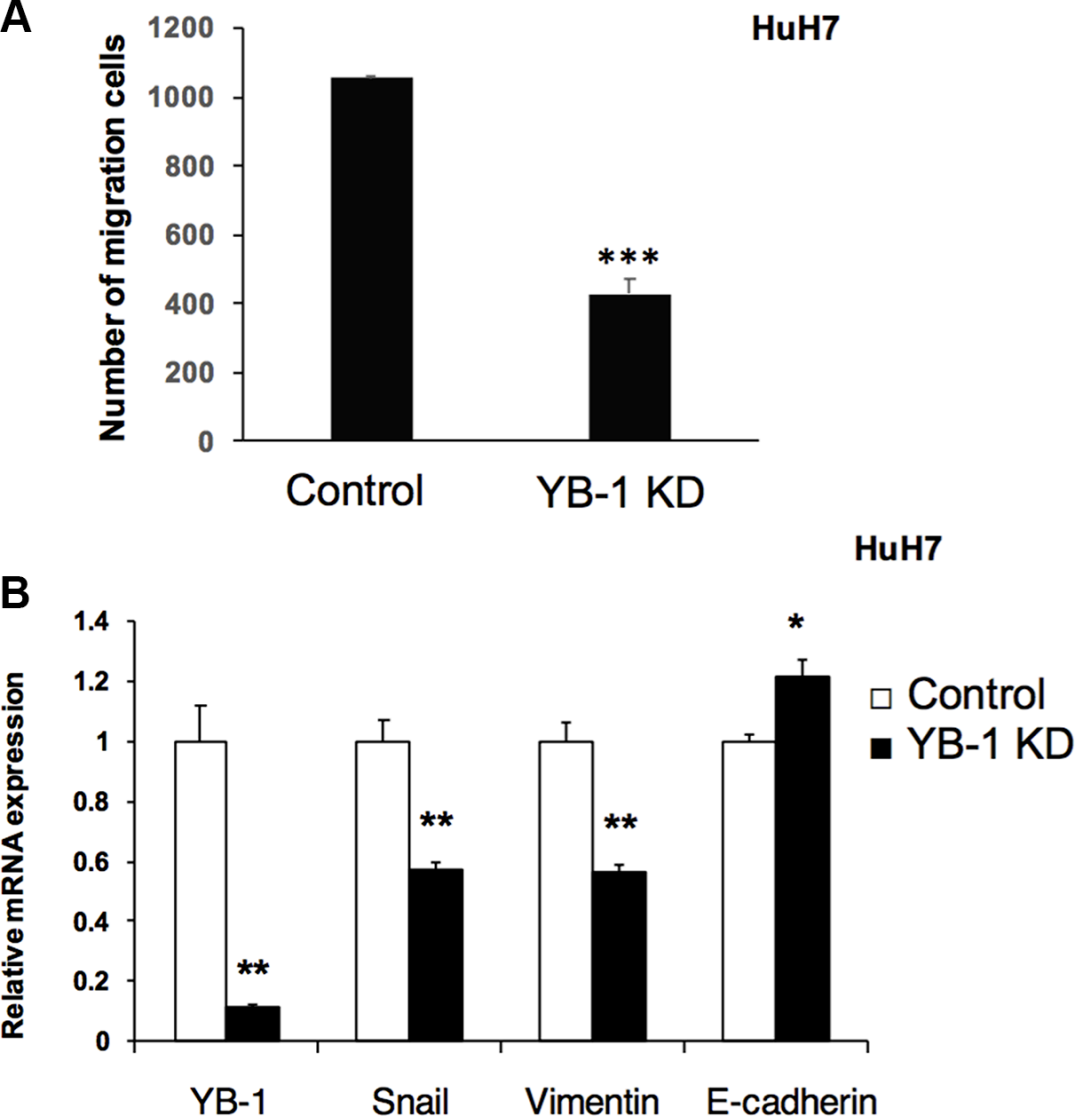
YB-1 Knockdown HCC cells decreased migration ability (**A**) Knock-down YB-1 inhibited migration ability of HuH7 cell. Cell migration was determined using Millipore Transwell chambers. The numbers of cells in five random microscopic fields were counted for each group. Data shown represent the means ± standard errors of the means (SEM) of data from at least 3 independent experiments. (**B**) EMT related genes were down-regulated in YB-1 knock-down HCC cells. The expression of snail and vimentin, the mesenchymal related genes, were down-regulated, and E-cadherin, an epithelial related gene, was up-regulated in YB-1 knock-down HuH7 cells. Expression levels were normalized to that of GAPDH. Each bar represents the means of three determinations ± SD. *p < 0.05, **p < 0.01 and ***p < 0.001 among the indicated groups.

The EMT occurs in wound healing, organ fibrosis, and initiation of metastasis during cancer progression. YB-1 has been reported to regulate several EMT-related genes and to promote the EMT process. Thus, we next examined whether YB-1 was involved in HCC migration using transwell migration assays. The result (Figure 4A) revealed that YB-1-knockdown cells exhibited reduced migration capacity compared with control cells. Moreover, YB-1 knockdown resulted in downregulation of the mesenchymal genes encoding Snail and vimentin and upregulation of the epithelial gene encoding E-cadherin (Figure 4B). These data indicated that YB-1 may be involved in the EMT in HCC cells.

### YB-1 increased drug resistance in HCC

**Figure 5:**
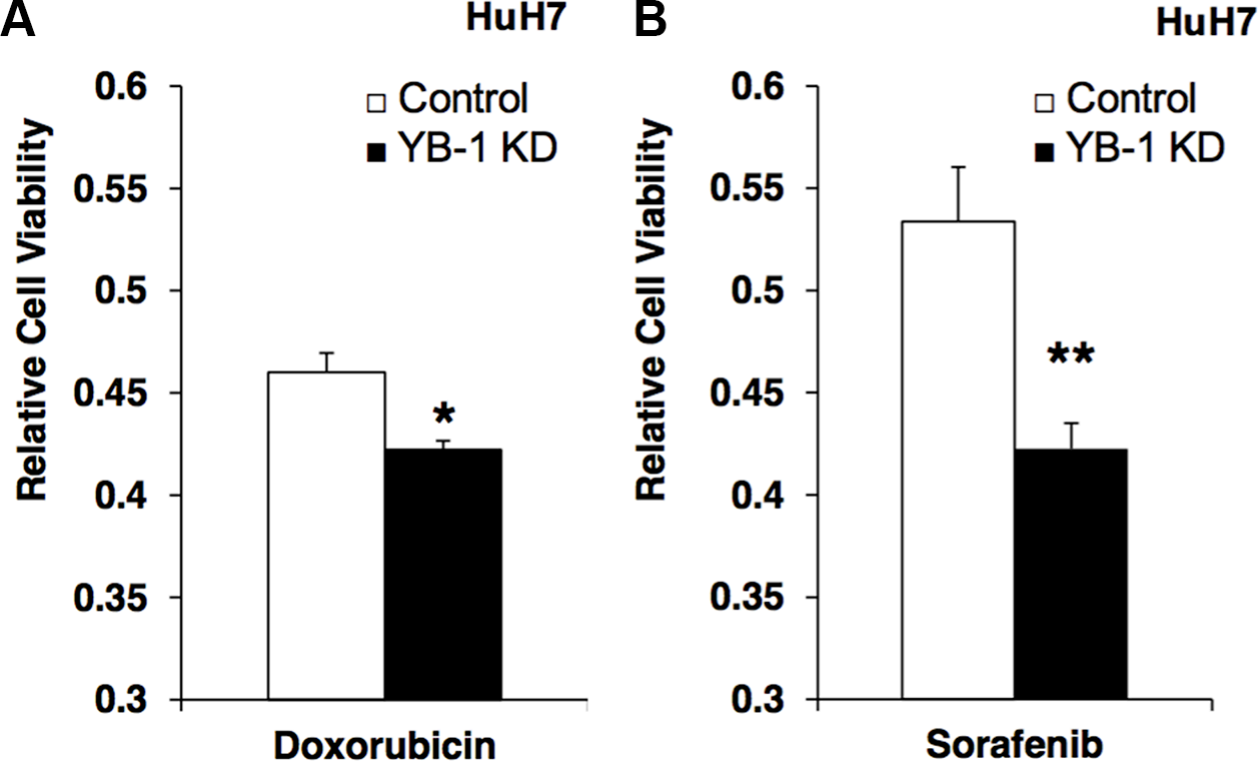
YB-1 Knockdown HCC cells decreased the drug resistance (**A**) Control and YB-1 KD HuH7 cells were treated with doxorubicin (0.15 μg/ml) or (**B**) sorafenib (0.15 mM) for 3 days in 3% FBS DMEM. Cell viability was assessed using the MTT assay. The cell viability of YB-1 KD cells was decreased compared with control cells. Each bar represents the means of three determinations ± SD. *p < 0.05; ** *p* < 0.01 among the indicated groups.

Chemotherapy has been widely used to treat cancers; however, the efficacy of different chemotherapy regimens is variable and may be related to the expression of multidrug-resistance genes. Although YB-1 has been shown to increase the expression of the ABC transporter MDR-1 in cancers, the role of YB-1 in drug resistance in HCC is unknown. Therefore, we next examined the effects of YB-1 knockdown on HCC cell numbers after treatment with doxorubicin and sorafenib, two drugs used to treat HCC in the clinical setting. In HCC cell culture, YB-1-knockdown cells exhibited decreased drug resistance against doxorubicin and sorafenib compared with control cells (Figure 5).

### YB-1 increased stemness and the cancer stem cell population in HCC

**Figure 6:**
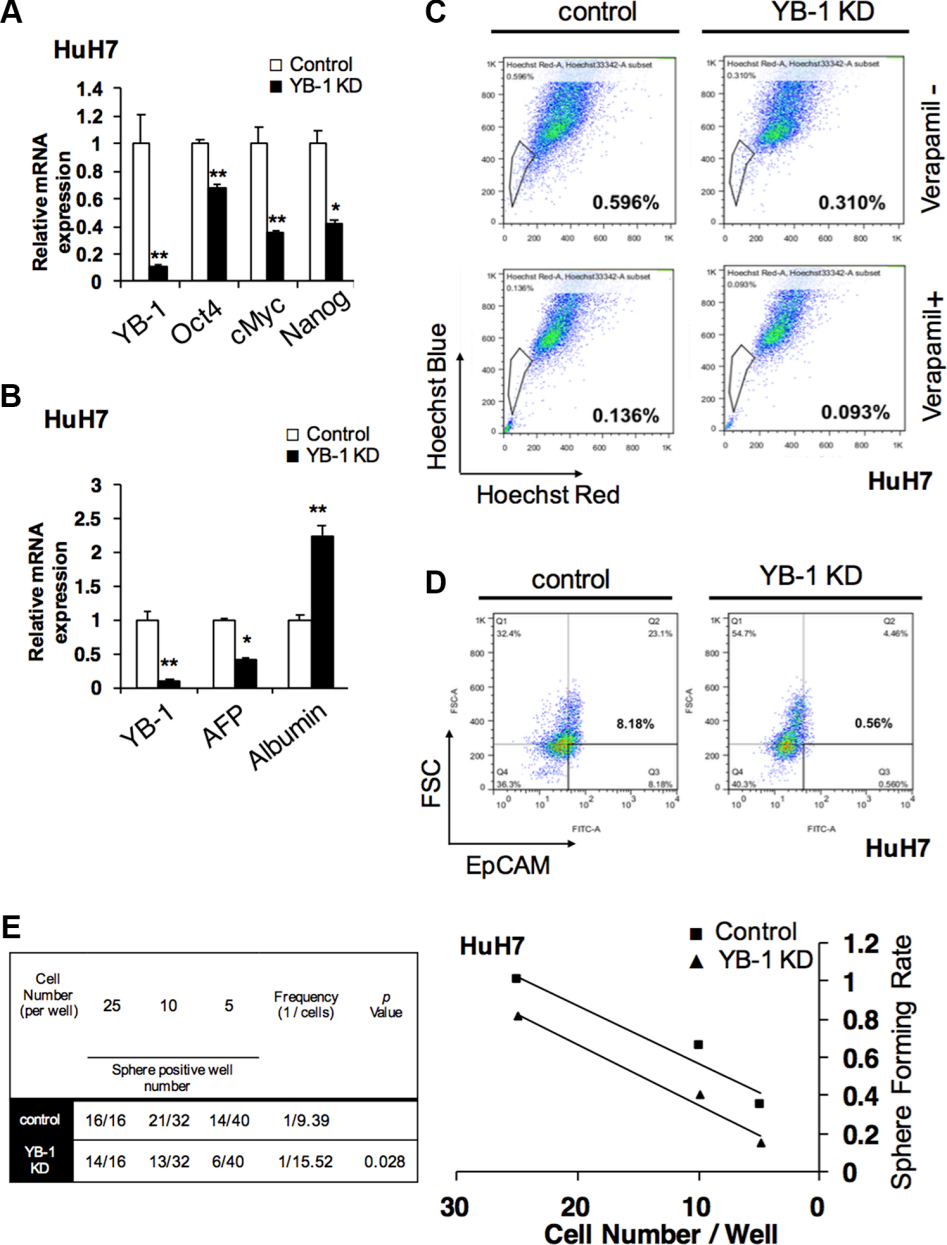
YB-1 promote HCC initiating cell properties and population (**A**) Stemness genes were downregulated and the differentiated gene was upregulated in YB-1 KD HCC cells. Stemness genes, Nanog, Oct4 and cMyc were decreased in YB-1 KD HuH7 cells. (**B**) Hepatic maturation marker gene, albumin, was up-regulated in YB-1 KD HuH7 cells. Relative expression of genes in HCC cells was analyzed by real-time PCR. Expression levels were normalized to that of GAPDH. Each bar represents the means of three determinations ± SD. *p < 0.05 and **p < 0.01 among the indicated groups. (**C**) Numbers of side-population (SP) cells were decreased in YB-1 KD HuH7 cells. The cells were detached, labeled with the Hoechst 33342 in the presence or absence of 50 μM verapamil and then analyzed by flow cytometry (BD Aria III). The SP cells disappeared in the presence of verapamil (lower panel). (**D**) Numbers of EpCAM positive cells were decreased in YB-1 KD HuH7 cells. The expression of HCC stem cell marker, EpCAM, bound with anti-EpCAM conjugated FITC antibodies was analyzed by flow cytometry (BD canto II). (**E**) HCC initiating cells frequency was declined in YB-1 KD HuH7 cells. Limiting dilution analysis of sphere formation was conducted to estimate the frequency of HCC initiating cells by fitting the single-hit Poisson model to the limiting-dilution data. The frequency of HCC stem cells was calculated using the extreme limiting dilution analysis platform.

YB-1 is upregulated in mouse fetal hepatoblasts. However, whether YB-1 affects the stemness and maturity of hepatoma cells is not known. As shown in Figure 6A, stemness-related genes, such as *Nanog*, *Oct4*, and *c-Myc*, were downregulated in YB-1-knockdown cells. The expression of alpha-fetoprotein, an immature molecular marker, was also decreased in YB-1-knockdown cells (Figure 6B). On the other hand, albumin, a marker of mature hepatocytes, was upregulated (Figure 6B). These results indicated that YB-1 may increase the stemness of hepatoma cells.

In order to determine whether YB-1 affected the population of HCC initiating cells, we investigated side-population and EpCAM+ hepatoma cells by flow cytometry. As shown in Figure 6C and 6D, the numbers of side-population cells and EpCAM+ cells were decreased after YB-1 knockdown.

### YB-1 was involved in HCC tumorigenesis

Sphere-forming assay has been widely used to identify stem cells based on the self-renewal and differentiation abilities of stem cells at the single cell level *in vitro*. In order to elucidate whether YB-1 was involved in the regulatory network of hepatic cancer initiating cells, extreme limiting dilution analysis (ELDA) [18], based on the sphere-forming assay *in vitro*, was used to analyze the cancer initiating cell frequency in hepatoma cells. As shown in Figure 6E and Supplementary Figure S2A, the frequency of cancer initiating cells was decreased, and the sphere formation ability was impaired in YB-1-knockdown cells. Taken together, these data suggested that YB-1 may promote the self-renewal abilities of cancer initiating cells to mediate tumorigenesis in HCC.

### YB-1 was involved in Wnt/β-catenin signaling pathway in HCC

**Figure 7:**
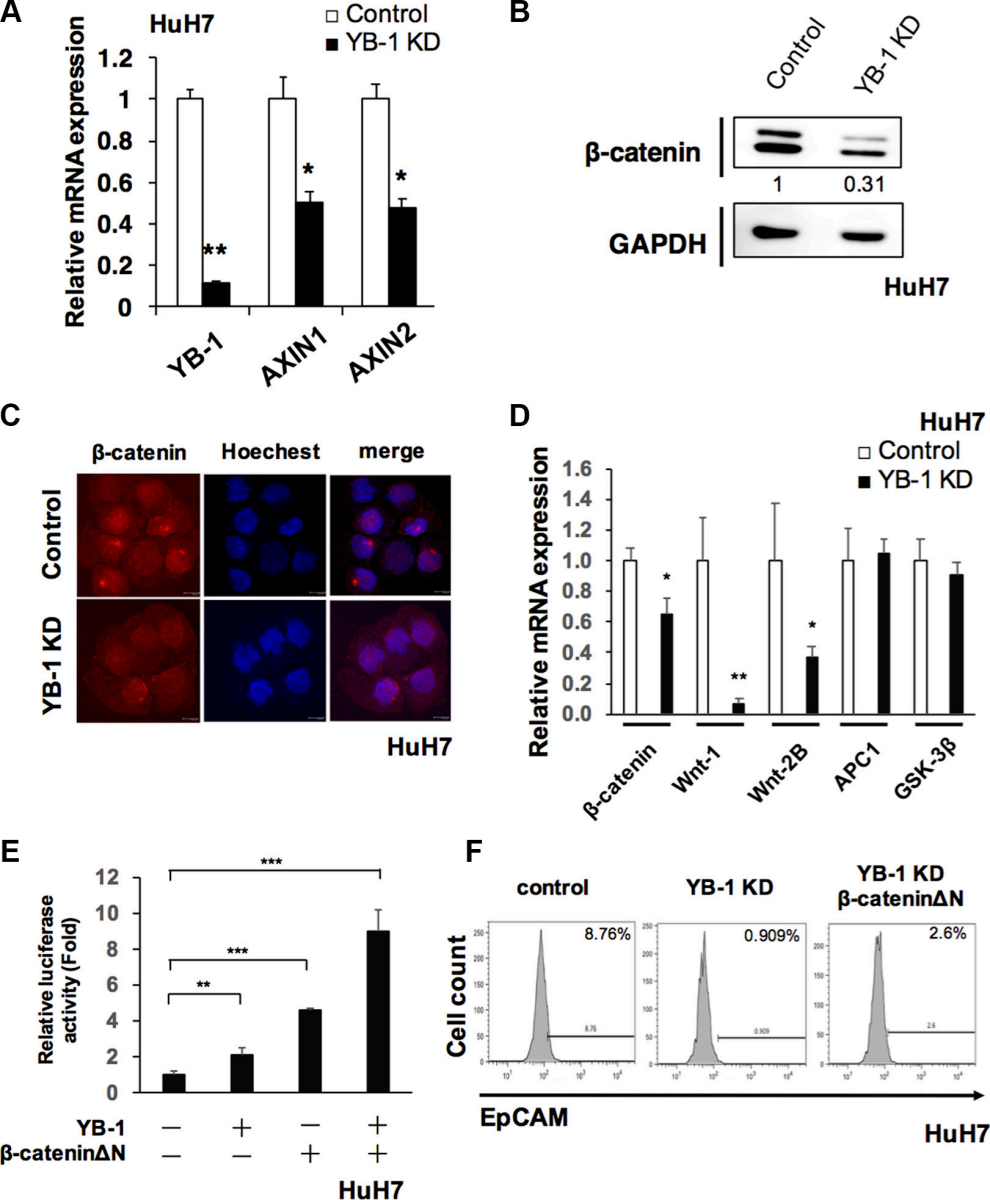
YB-1 promoted Wnt/β-catenin signaling (**A**) Wnt/β-catenin signaling target genes, Anix1 and Anix2 were downregulated in YB-1 KD HuH7 cells. Relative expression of genes in HuH7 cells was analyzed by real-time PCR. Expression levels were normalized to that of GAPDH. Each bar represents the means of three determinations ± SD. *p < 0.05, **p < 0.01 among the indicated groups. (**B**) β-catenin was downregulated in YB-1 KD HuH7 cells. Protein level of β-catenin was detected by western blot. The protein expression was normalized to GAPDH. (**C**) Immunofluorescence staining of β-catenin in control and YB-1 KD HuH7 cells. (**D**) Wnt ligands were down-regulated in YB-1 KD HuH7 cells. Relative expression of genes in HuH7 cells was analyzed by real-time PCR. Expression levels were normalized to that of GAPDH. Each bar represents the means of three determinations ± SD. *p < 0.05, **p < 0.01 among the indicated groups (**E**) TOP-Flash luciferase reporter assay showed the activation of Wnt signaling in HuH7 cells. Overexpression of YB-1and β-cateninΔΝ increased TOP-Flash promoter activity. Each bar represents the means of three determinations ± SD. *p < 0.05, **p < 0.01, ***p < 0.001 among the indicated groups. (**F**) β-cateninΔΝ rescued EpCAM+ cell population that decreased in YB-1 KD HuH7 cells.

Wnt/β-catenin signaling pathway plays an important role in HCC initiating cells, which maintains HCC initiating cells stem cell properties and is involved in HCC tumorigenesis [19, 20]. In Figure 7A, knockdown of YB-1 suppressed Wnt signaling target genes, *Axin1* and *Axin2*. The protein level of β-catenin was also downregulated in both YB-1 KD hepatoma cells and YB-1 KD sphere cells (Figure 7B and Supplementary Figure S2B). YB-1 knockdown hepatoma cells exhibited reduced nuclear translocation of β-catenin (Figure 7C). We also demonstrated that the expression of WNT-1 and WNT-2B were downregulated in YB-1 KD cells (Figure 7D). Moreover, overexpression of YB-1 increased the TOP-Flash transcriptional activity (Figure 7E).

In order to realize whether YB-1 promoted stemness via Wnt/β-catenin pathway, the rescue experiment was carried out using the active form β-catenin (β-cateninΔN, lack of the phosphorylation domain of GSK-3β). Overexpression of β-cateninΔN in the YB-1 KD cells could rescue the HCC initiating cell (EpCAM+ cell) population and the expression of stemness genes (Figure 7F and Supplementary Figure S3). Overexpression of both YB-1 and β-cateninΔN in hepatoma cells could additionally increase the TOP-Flash transcriptional activity (Figure 7E). Taken together, YB-1 maintained the stemness feature of HCC initiating cells probably via Wnt/β-catenin signaling.

### Cellular localization of YB-1 in HCC initiating cells

**Figure 8:**
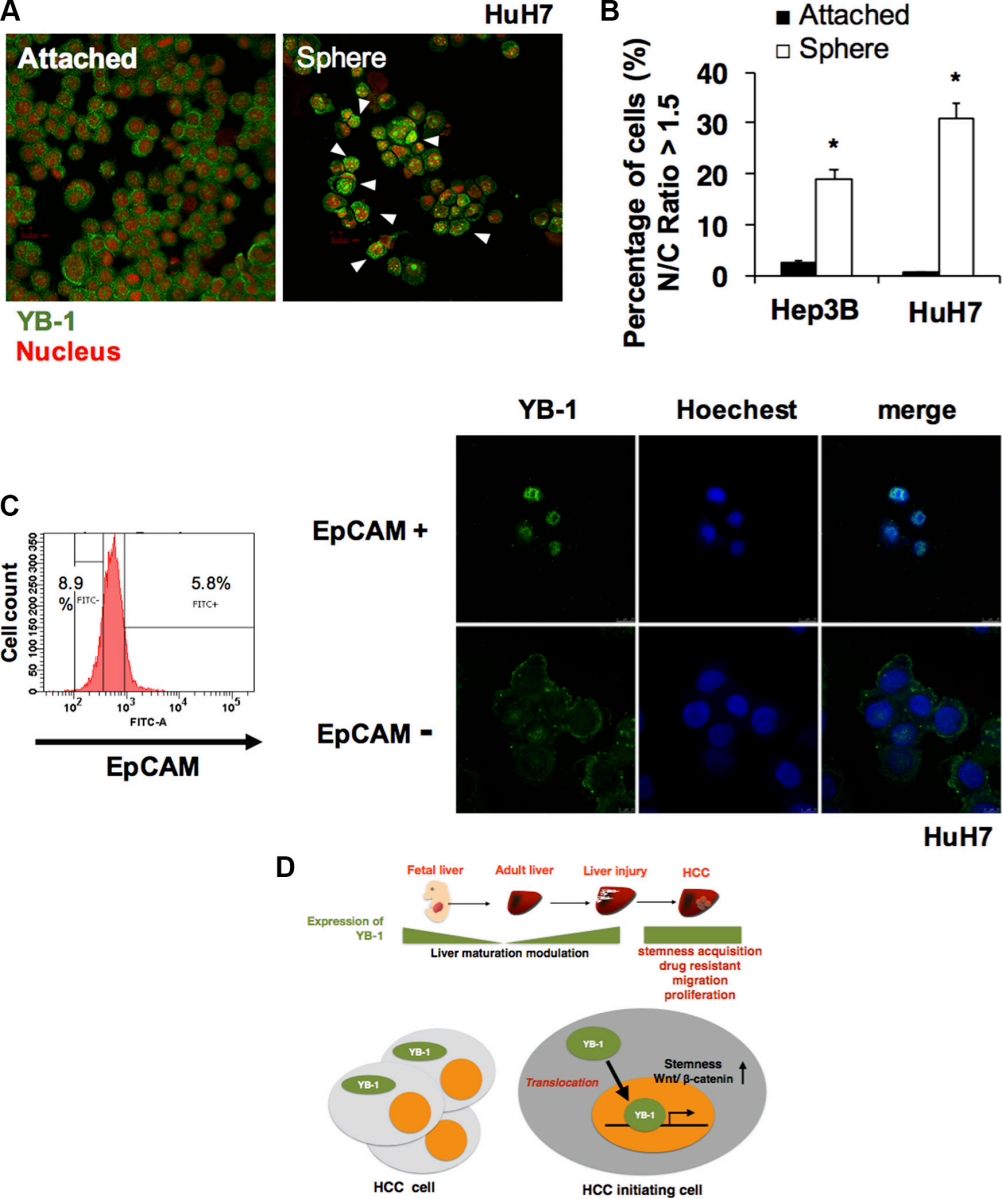
YB-1 translocated into nucleus in HCC initiating cells (**A**) Immunofluorescence staining of YB-1 in attached 2D HuH7 cells and HuH7 sphere cells. YB-1 was mainly sub-localized in the nucleus of sphere cells. 2D attached HCC cells and sphere cells were stained for YB-1 (green). Nucleus was stained by PI (red). Representative images are shown in (A). (**B**) The nucleus/cytoplasm ratio of YB-1 distribution in HCC cells was quantified by high content cell analyzer (GE IN CELL 2000). The percentage of HCC cells with nucleus/cytoplasm ratio of YB-1 localization more than 1.5 was calculated. Each bar represents the means of three determinations ± SD. (*) *P* < 0.05 among the indicated groups. (**C**) YB-1 was mainly localized in the nucleus of EpCAM+ cells. (**D**) Fetal liver protein YB-1 was upregulated again in injured liver and HCC. YB-1 promoted HCC progression and nuclear translocation of YB-1 in HCC initiating cells may increase stem cell features via transcriptional regulation.

YB-1 modulates the expression of genes via DNA transcription and RNA translation depending on its subcellular localization. In human HCC, expression of YB-1 in both the nucleus and cytoplasm of HCC cells results in poor clinical outcomes compared with that observed when YB-1 is expressed only in the cytoplasm. To determine whether there was a subgroup of HCC cells with predominantly nuclear expression of YB-1, we investigated the subcellular localization of YB-1 in HCC cells by high-content analysis. As shown in Figure 8, YB-1 was mainly expressed in the cytoplasm of HCC cells in the attached culture, whereas YB-1 was predominantly present in the nucleus of sphere cells and EpCAM+ cells (Figure 8A–8C). Moreover, nuclear localization of β-catenin was also observed in the EpCAM+ cells (Supplementary Figure S4) These results may explain the two different immunohistochemical expression patterns of YB-1 in human HCC and revealed that YB-1 exerted its specific functions in cancer initiating cells via DNA transcription.

## DISCUSSION

YB-1 is a pleiotropic molecule that binds DNA and RNA to regulate genes at the transcriptional level in the nucleus and translational level in the cytoplasm, respectively. In the present study, we found that YB-1 was expressed in HCC cell lines. After loss of YB-1 expression, HCC cells exhibited decreased expression of proliferation markers, such as cyclin A and cyclin B, increased expression of the tumor-suppressor gene *p53*, and inhibition of cell growth. The expression of cell cycle genes was increased in mouse livers through the hydrodynamic gene delivery method, and YB-1 was found to promote cell proliferation *in vitro* and *in vivo*. Our previous study showed that YB-1 is upregulated during liver development and liver regeneration, in which hepatocytes require vigorous cell proliferation. Compared with mature hepatocytes, hepatocytes present during liver development and regeneration or in HCC are in an immature state in which physiological functions are suppressed. Moreover, our previous study showed that YB-1 inhibits the expression of CPS1 to impair the ammonia metabolism [10]. Thus, taken together, these data suggested that YB-1 may regulate the balance between cell proliferation and differentiation during liver development and injury.

HCC is a heterogeneous disease. Increasing evidence has shown that a subgroup of cancer initiating cells has stem cell-like properties, such as self-renewal, sphere-forming ability, and production of heterogeneous progeny. These cancer initiating cells possess greater chemoresistance/radioresistance and tend to show increased invasiveness and migration capacity through promotion of the EMT, which is associated with cancer recurrence and metastasis. Here, we showed that YB-1 was highly expressed in cancer initiating cells in HCC cells. Silencing of YB-1 resulted in decreased HCC migration, increased EMT-related gene expression, and reduced chemoresistance. Furthermore, the ratio of cancer initiating cells in HCC was decreased, and tumorigenesis and initiating cell frequency in HCC cells was lower, as shown by extreme limiting dilution analysis following knockdown of YB-1. These results implied that YB-1 acted as a marker of HCC and played essential roles in maintaining the numbers of HCC initiating cells and the tumorigenic capacity. On the other hand, Wnt/β-catenin signaling plays a key role in stem cell feature maintenance in HCC [20]. According to the ChIP-on-chip data in breast cancer by Dr. Dunn et al. [21], YB-1 might be recruited on the promoter of *wnt1, wnt2b, wnt4, wnt3A, wnt5b, wnt10a*, *wnt11* and *wnt16* genes. Here, we found that knockdown of YB-1 inhibited the expression of WNT-1 and WNT-2B. However, the expression of other Wnt family was not changed or undetectable. Moreover, silencing of YB-1 inhibited the nuclear translocation of β-catenin in HCC cells. Overexpression of active form β-cateninΔΝ in the YB-1 KD cells rescued the HCC initiating cell population and tumor stemness. These data suggested that YB-1 may promote Wnt/β-catenin signaling via upregulation of WNT-1, WNT-2B and β-catenin. Moreover, YB-1 may be associated with the progression of HCC. Consistent with our previous study, YB-1 was shown to be upregulated in the fetal and regenerating liver and was a marker of liver stem cells; high expression of YB-1 in the HCC subpopulation may be correlated with stemness. In addition to liver development, YB-1 plays important roles in the development of the brain [22]. Recent research has revealed that YB-1 is also a marker for neural stem cells and is expressed in tumor-initiating cells in the brain, which participate in the development of glioblastoma [23]. Additionally, YB-1 is a key factor contributing to the increase in stemness. For example, YB-1 increases the expression of stem cell marker proteins, such as CD44 and CD49f, in breast cancer [6]. Thus, YB-1 may play essential roles in tumor initiation and development.

Clinical analysis of cBioportal database showed that YB-1 was frequently expressed in human HCC and associated with poor survival of patients (Figure 1A). Yasen's clinical data also revealed the same tendency [11]. Moreover, YB-1 was detected in the cytoplasm or in both the cytoplasm and nucleus. Patients with HCC harboring YB-1 localized in the nucleus tended to have poorer prognoses and lower survival rates. However, the relevance of the subcellular localization of YB-1 in HCC remains unclear. Previous studies have shown that YB-1 translocates into the nucleus to regulate transcription under genotoxic stress, such as UV or chemotherapeutic drugs [24, 25]. For example, YB-1 modulates the expression of the *MDR-1* gene at the transcription level [4]. In our study, YB-1 was found to induce the expression of EMT- and stemness-related genes in HCC cells. We also found that most HCC initiating cells (EpCAM+ cells) or sphere cells expressed YB-1 in the nucleus. These data suggested that nuclear YB-1 may drive HCC cells to obtain stem cell-like properties, thereby maintaining the number of HCC initiating cells and increasing chemoresistance and invasive capacity. This may explain why HCC patients with nuclear YB-1 have poor disease-free survival rates [11]. Furthermore, recent studies have shown that recurrent ovarian cancer cells tend to have increased nuclear YB-1 [26]. Thus, nuclear YB-1 may be related to higher recurrence rates and common cancer stem cell properties.

Liver carcinogenesis is a progressive process involving cellular transformation, cancer cell proliferation, and metastasis; this process may be reflected in the differential expression and subcellular localization of YB-1. YB-1 is induced transiently in hepatocytes to increase proliferative capacity and repair the liver when the liver is injured or subjected to environmental stress. Permanent YB-1 expression may be one of the factors driving the initiation of hepatocellular carcinogenesis. YB-1-expressing cells may further obtain stem cell-like properties when YB-1 translocates into the nucleus under niche stimulation (Figure 8D). However, the mechanisms through which YB-1 is induced are unclear. Elucidation of the regulatory mechanisms of YB-1 expression and the relevance of its nuclear localization in HCC may improve our understanding of HCC tumorigenesis and progression and provide a novel therapeutic target in HCC. In addition, owing to its potential roles in chemoresistance, YB-1 could be a potential adjuvant target molecule combined with conventional chemotherapy to elevate the chemosensitivity of HCC.

## MATERIALS AND METHODS

### Cell lines

HuH7, HepG2, JHH5 and Hep3B HCC cell lines were cultured in Dulbecco's modified Eagle's medium (DMEM, Life Technologies) supplemented with 10% FBS (GE Healthcare) and 1% penicillin/streptomycin/glutamine (PSG, Life Technologies) and maintained at 37°C in a humidified incubator with 5% CO_2_. The proliferative response of YB-1 knockdown HCC cells was examined by the BrdU Cell Proliferation Kit (Millipore) according to the manufacturer's procedures.

### Animals

C57BL/6J mice were obtained from CLEA Japan, Inc. The experiments were performed according to the guideline set by the institutional animal care and use committee of the University of Tokyo. Hydrodynamic gene delivery was allowed the standard procedure of TransIT-EE Delivery Kit (Mirus Bio). Twenty micrograms of purified plasmid (pLIVE-YB-1 or pLIVE-LacZ control plasmid) was dissolved in 1.8 ml of TransIT-EE Delivery Solution (Mirus Bio) and then was injected via mouse tail vein.

### Sphere formation assay

To obtain individual single cell, HuH7 or Hep3B cells were treated with 0.5% trypsin. Cells were then resuspended in DMEM/F12 medium (Life Technologies) containing B27 (Life Technologies), human recombinant EGF (20 ng/ml; Pepro Tech), bFGF (20 ng/ml; Pepro Tech), plated at a density of 1 × 10^3^ live cells/ml medium on ultra-low attachment dish or plate and cultured for 6 days. For limited dilution assay, cells were seeded at different densities, including 1 × 10^3^ cells/mL, 5 × 10^2^ cells/mL, 2.5 × 10^2^ cells/mL, and 1 × 10^2^ cells/mL for 16 wells, 32 wells, 48 wells, and 96 wells in ultra-low attachment 96 well-dish. These cells were cultured for 8 to 10 days. Spheres were detected by staining with Hoechst 33342 at 37°C for 30 to 40 minutes and analyzed with In Cell 2000 Analyzer (GE Healthcare). The groups were compared by Mann Whitney test, *P* < 0.05 was considered to be significantly different. For limited dilution assay, data were analyzed by webtool at http://bioinf.wehi.edu.au/software/elda/, *P* < 0.05 was considered to be significantly different.

### RNA extraction and quantitative PCR analysis

The total RNA was extracted by Tripure (Roche) following standard protocol. Total mRNA was first reverse transcribed into cDNA using High-Capacity cDNA Reverse Transcription Kit (Applied Biosystem) according to the manual protocol. qPCR was performed using iQ SYBR green detection system in Bio-Rad. The expression level of target genes was normalized to GAPDH expression. The primers used for qPCR were shown in Supplementary Table S2.

### Stable clone establishment

The pLKO.1-YB-1 shRNA plasmids purchased from RNAi Core, Academia Sinica, was transfected into HuH7 with Jet-prime transfection reagent (Polyplus). After 2days, cells were selected by 10% FBS DMEM containing 1 μg/ml puromycin. 14 days later, single colonies were isolated and amplified. Knock down efficiency was checked by qPCR and Western blot. Transient siRNA knockdown was performed by BLOCK-iT™ RNAi system (ThermoFisher), The sequences were shown in Supplementary Table S2. β-cateninΔΝ was overexpressed to YB-1 KD HuH7 cells by lentiviral system. HEK293T cells were transfected with three lentivirus-packaging plasmids (pMDLg/pRPE, pMD2G, pRSV-Rev, Addgene) and the lentivrial pSLIK-β-cateninΔΝ by JetPRIME (Polyplus-transfection) reagent following the manufacturer's protocol. After 48 hrs incubation, supernatants containing lentivirus particles were collected by centrifugation with 8.5% polyethylene glycol 6000 and 0.3 M sodium chloride.

### Colony formation assay

HuH7 or Hep3B cells were treated with 0.5% trypsin. Single cells were then resuspended in 3% FBS DMEM, plated on cell culture dish and cultured for 14 days. For counting colonies, cells were washed twice with cold PBS, fixed with cold methanol for 5 minutes, stained with 0.5% crystal violet and destained with PBS. Then, pictures were taken and analyzed by ImageJ software.

### Drug resistance assay

1 × 10^5^ Cells per well were seeded on 96-well plate. After 24 hours., cells were treated with 150 ng/ml Doxorubicin or 0.15 mM Sorafenib for 3 days. Cell viability was measured by the MTT assay according to the manual protocol.

### Flow cytometry and cell sorting

Primary conjugated antibody (or isotype control) was added into each sample and incubated for an hour at 4°C. Cells were then stained with propidium iodide for 10 minutes (PI, Life Technologies). BD FACSCantoII and BD AriaIII were used to analyze the expression of markers and for cell sorting, respectively. The antibodies used in this study and its dilution condition was shown in Supplementary Table S1. Side Population was analyzed using flow cytometry. The cells were detached from the dishes with Trypsin- EDTA (Invitrogen) and suspended at 1 × 10^6^ cells/mL in PBS solution supplemented with 3% fetal calf serum. These cells were then incubated at 37°C for 90 minutes with 20 g/mL Hoechst 33342 (Sigma), either alone or in the presence of 50 mol/L verapamil (Sigma). After incubation, 1 g/mL propidium iodide (BD Pharmingen, San Diego, CA) was added and then filtered through a 40 μm cell strainer (BD Falcon) to obtain single-suspension cells. Cell analysis was performed using BD AriaIII.

### Transwell migration assay

Cells were starved with serum-free DMEM medium for 3 hours. After starvation, cells were harvested and resuspended by trypsinization. In the migration assay, 24-well culture plates were divided into upper and lower wells by transwell inserts (BD Bioscience). The upper surface of the transwell was loaded with 2 × 10^5^ cells in 300 μL serum-free DMEM medium, while the lower well contained 500 μL DMEM with 10% FBS. Following 6 hours of incubation, the migrated cells on the bottom surface were fixed with methanol for 10 minutes and counted after staining with crystal violet for 1 hour.

### Luciferase reporter assay

The TOP-Flash luciferase reporter construct contains 3 copies of the Tcf/LEF-binding site (AAGATCAAAGGGGGT) upstream of a TK minimal promoter. HuH7 were transfected with TOP-Flash and pmCherry-C1 (Clontech) plasmid by JetPRIME reagent (Polyplus-transfection) following the manufacturer's protocol. After pME-YB-1 and β-cateninΔΝ plasmids transfection for 48 hours, the cells were harvested for luminescence measurement. Fluorescence intensity of mCherry was measured for internal control.

### Immunofluorescence staining

Liver tissue was embedded in OCT (Sakura Finetek) and cryosectioned into 8 μm thick sample using Leica CM 1900 (Leica). The HCC cells were concentrated on the slide glass by Cytospin (Shandon). The samples were fixed with 4% paraformaldehyde (Sigma Aldrich) and permeabilized with 0.1% saponin (Sigma) for 15 minutes and 15 minutes, respectively. After the removal of culture medium, cells were fixed with 4% paraformaldehyde and permeabilized with 0.1% Triton X100 (Riedel-de-Haën) for 7 minutes and 15 minutes, respectively. Then the samples were blocked with 4% fetal bovine serum (FBS, diluted in PBS), stained by primary antibody solution at 4°C for overnight, secondary antibody at room temperature for an hour, and stained with Hoechst 33342 for 10 minutes before analyzed by fluorescence microscope or high content IN Cell Analyzer (GE Healthcare) and confocal fluorescence microscopy (Leica). The antibodies used in this study and its dilution condition was shown in Supplementary Table S1.

### Statistics

qRT-PCR data in the bar charts represent means ± SEM and were obtained from average data of three independent experiments. Statistical significance was calculated using a two-tailed Student’s-test. Differences with the *P* value of less than 0.05 were considered significant, and those with *P* value of less than 0.01 were considered really significant.

## SUPPLEMENTARY MATERIALS FIGURES AND TABLES





## Abbreviations

CD: cluster of differentiation
CPS1: Carbamoyl phosphate synthetase I
DNA: deoxyribonucleic acid
ELDA: extreme limiting dilution analysis
EMT: epithelial-mesenchymal transition
HCC: hepatocellular carcinoma
MDR-1: multidrug resistance gene
miRNAs: microRNAs
Nanog: Nanog Homeobox
Oct4: octamer-binding transcription factor 4
PCNA: proliferating cell nuclear antigen
RNA: Ribonucleic acid
SP: side-population
YB-1: Y-box binding protein-1;Wnt: Wingless-type MMTV integration site family member.
