# Supplementary material for: Y-box binding protein-1 promotes hepatocellular carcinoma-initiating cell progression and tumorigenesis via Wnt/β-catenin pathway
Source: Oncotarget. 2016 Dec 1;8(2):2604–16. doi: 10.18632/oncotarget.13733 (PMC5356827; doi:10.18632/oncotarget.13733)
Supplement: Supplementary file 1 [file oncotarget-08-2604-s001.pdf]

Y-box binding protein-1 promotes hepatocellular carcinoma-initiating cell progression and tumorigenesis via wnt/β-catenin pathway

Supplementary Materials

Supplementary Table S1: List of antibodies

Antibody	Manufacturer	Catalog Number	Application	Dilution Fold
**Primary Antibodies**				
Anti-YB-1	Abcam	ab12148	IF	1/300
Anti-YB-1	Abcam	ab12148	IB	1/10000
Anti-Cyclin A2	Abcam	Ab-7	IB	1/5000
Anti-Cyclin B2	Abcam	Ab-3	IB	1/5000
Anti-P53	Santa Cruz	SC-126	IB	1/1000
Anti-GAPDH	Millipore	MAB374	IB	1/10000
Anti-β-catenin	Abcam	Ab-2365	IF	1/300
Anti-β-catenin	Abcam	Ab-2365	IB	1/10000
Anti-EPCAM	Biolegend	324203	FC	1/250
Mouse Isotype IgG	eBioscience	11-4714	FC	1/250
**Secondary Antibodies**				
Anti-rabbit AlexaFlouro488	Life Technologies	A21206	IF	1/200
Anti-mouse HRP	Jackson ImmunoResearch	115-035-003	IB	1/10000
Anti-rabbit AlexaFlouro555	Life Technologies	A21206	IF	1/200
Anti-rabbit HRP	Jackson ImmunoResearch	111-035-003	IB	1/10000

Supplementary Table S2: List of qPCR primers and oligonucleotides sequences of siRNA.

See Supplementary_Table_S2

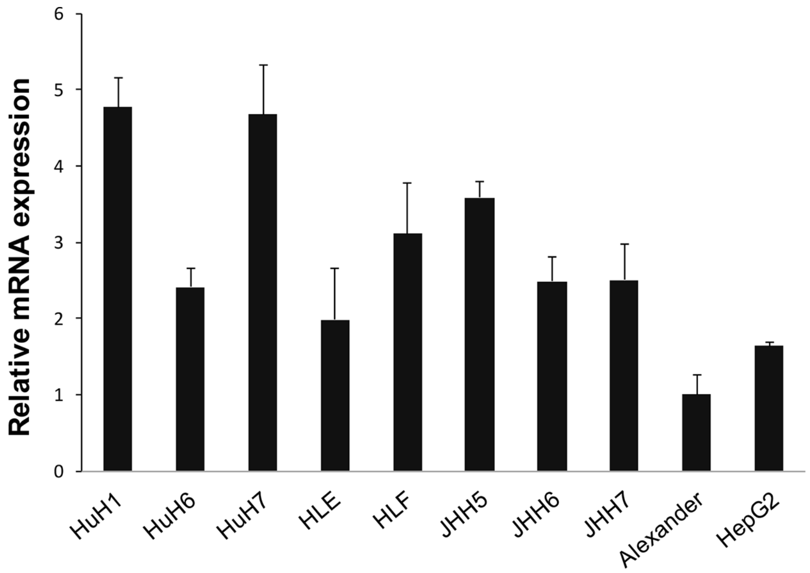

Supplementary Figure S1: The expression of YB-1 in hepatoma cell lines. Relative expression of YB-1 in hepatoma cell lines were analyzed by real-time PCR. Expression levels were normalized to that of GAPDH. Each bar represents the means of three determinations ± SD.

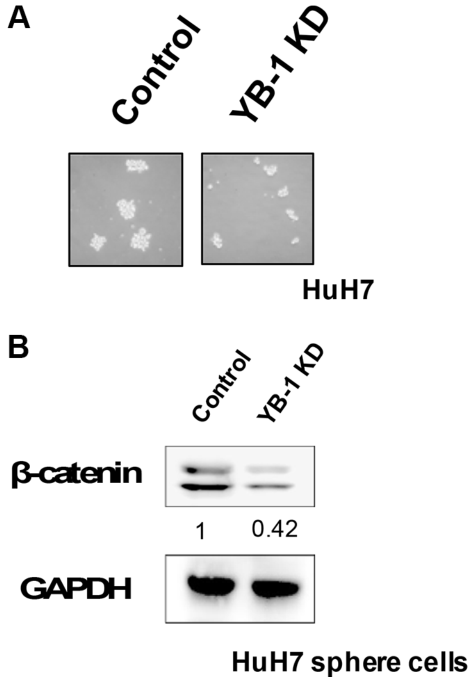

Supplementary Figure S2: Sphere forming of HuH7 cells. (**A**) HuH7 cells were cultivated on ultra-low attachment dish for 6 days (**B**) The protein expression level of β-catenin in sphere cells was measured by western blot. Relative expression level was normalized to GAPDH.

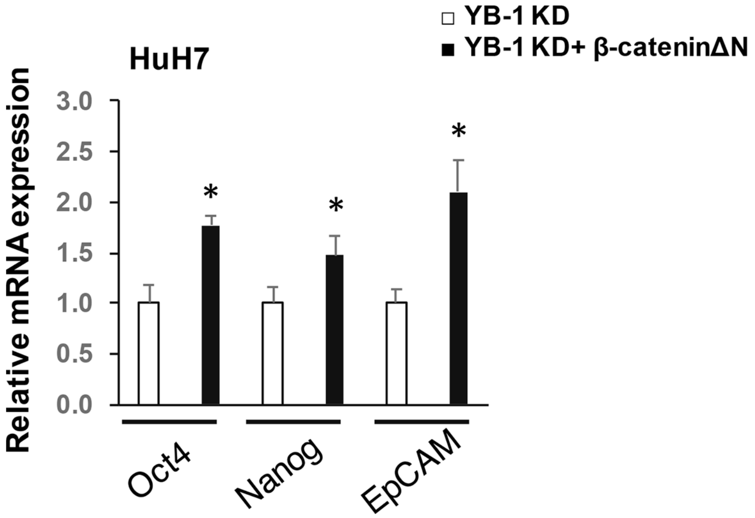

Supplementary Figure S3: The expression level of Oct4, Nanog and EpCAM were upregulated in β-cateninΔN overexpressed YB-1 KD HuH7 cells. Relative expression of genes in HuH7 cells was analyzed by real-time PCR. Expression levels were normalized to that of Gapdh. Each bar represents the means of three determinations ± SD.*$p < 0.05$ among the indicated groups.

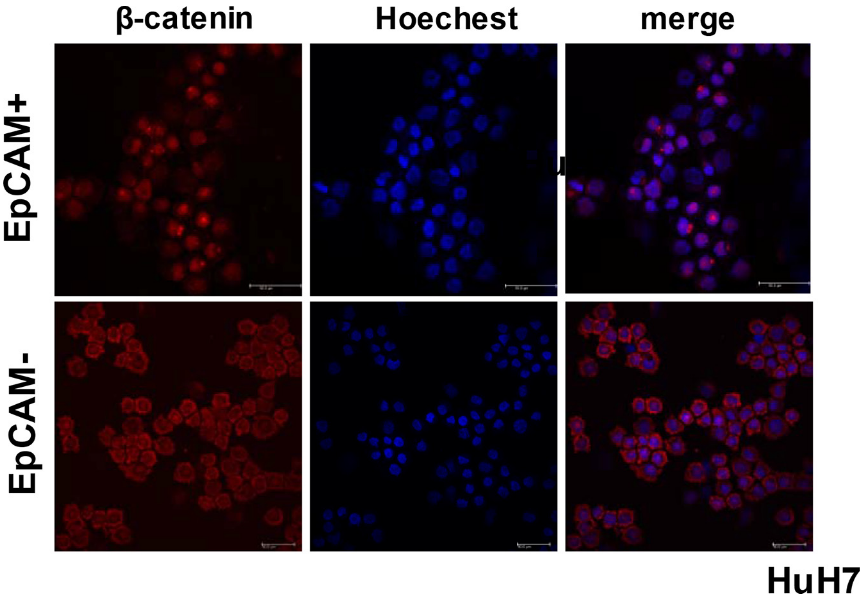

Supplementary Figure S4: Immunofluorescence staining of β-catenin in EpCAM+ and EpCAM- cells of HuH7.
