# Supplementary material for: Y-box binding protein-1 promotes hepatocellular carcinoma-initiating cell progression and tumorigenesis via Wnt/β-catenin pathway
Source: Oncotarget. 2016 Dec 1;8(2):2604–16. doi: 10.18632/oncotarget.13733 (PMC5356827; doi:10.18632/oncotarget.13733)
Supplement: Supplementary file 2 [file oncotarget-08-2604-s002.docx]

Supplementary Table 2. List of qPCR primers and oligonucleotides sequences of siRNA

| **Gene** | **Primers** | |
| --- | --- | --- |
| GAPDH | Forward | GGAGCGAGATCCCTCCAAAAT |
|  | Reverse | GGCTGTTGTCATACTTCTCATGG |
| β-Actin | Forward | CATGTACGTTGCTATCCAGGC |
|  | Reverse | CTCCTTAATGTCACGCACGAT |
| YB-1 | Forward  (human) | TAGACGCTATCCACGTCGTAG |
|  | Reverse  (human) | CCCCACTCTCACTATTCTGGT |
|  | Forward  (mouse) | CCCTTACAGACCACGATTCC |
|  | Reverse  (mouse) | GGCGATACCGACGTTGAG |
| Albumin | Forward | GAGACCAGAGGTTGATGTGATG |
|  | Reverse | AGTTCCGGGGCATAAAAGTAAG |
| AFP | Forward | CTTTGGGCTGCTCGCTATGA |
|  | Reverse | GCATGTTGATTTAACAAGCTGCT |
| OCT4 | Forward | GACAGGGGGAGGGGAGGAGCTAGG |
|  | Reverse | CTTCCCTCCAACCAGTTGCCCCAAAC |
| cMYC | Forward | GTCAAGAGGCGAACACACAAC |
|  | Reverse | TTGGACGGACAGGATGTATGC |
| Nanog | Forward | CGTGTGAAGATGAGTGAAACTGA |
|  | Reverse | CTCGCTGATTAGGCTCCAAC |
| SNAIL1 | Forward | TCGGAAGCCTAACTACAGCGA |
|  | Reverse | AGATGAGCATTGGCAGCGAG |
| Vimentin | Forward | AGTCCACTGAGTACCGGAGAC |
|  | Reverse | CATTTCACGCATCTGGCGTTC |
| E-cadherin | Forward | ATTTTTCCCTCGACACCCGAT |
|  | Reverse | TCCCAGGCGTAGACCAAGA |
| Wnt-1 | Forward | CGATGGTGGGGTATTGTGAAC |
|  | Reverse | CCGGA TTTTG GCGTA TCAGAC |
| Wnt-2B | Forward | CGGGACCACACCGTCTTTG |
|  | Reverse | GCGAGTAATAGCGTGGACTAC |
| CTNNB | Forward | CATCTACACAGTTTGATGCTGCT |
|  | Reverse | GCAGTTTTGTCAGTTCAGGGA |
| APC1 | Forward | AAAATGTCCCTCCGTTCTTATGG |
|  | Reverse | CTGAAGTTGAGCGTAATACCAGT |
| Cyclin D1 | Forward  (mouse) | GCGTACCCTGACACCAATCTC |
|  | Reverse  (mouse) | CTCCTCTTCGCACTTCTGCTC |
| Cyclin A2 | Forward  (human) | TAGCTGCTCCAACAGTAAATCAG |
|  | Reverse  (human) | AGGTATGGGTCAGCATCTATCAA |
|  | Forward  (mouse) | GCCTTCACCATTCATGTGGAT |
|  | Reverse  (mouse) | TTGCTGCGGGTAAAGAGACAG |
| Cyclin B1 | Forward  (human) | TTGGGGACATTGGTAACAAAGTC |
|  | Reverse  (human) | ATAGGCTCAGGCGAAAGTTTTT |
|  | Forward  (mouse) | AAGGTGCCTGTGTGTGAACC |
|  | Reverse  (mouse) | GTCAGCCCCATCATCTGCG |
| PCNA | Forward | ACACTAAGGGCCGAAGATAACG |
|  | Reverse | ACAGCATCTCCAATATGGCTGA |
| P53 | Forward | GAGGTTGGCTCTGACTGTACC |
|  | Reverse | TCCGTCCCAGTAGATTACCAC |
| EpCAM | Forward | ATAATCGTCAATGCCAGTGTA |
|  | Reverse | TTTGCTCTTCTCCCAAGTTT |
| GSK-3β | Forward | AGACGCTCCCTGTGATTTATGT |
|  | Reverse | CCGATGGCAGATTCCAAAGG |
| **siRNA** | | **Sequence** |
| siYB-1#1 | | AAAGGGUGCGGAGGCAGCAAAUGUU |
| siYB-1#2 | | CAAGGUAGACCAGUGAGACAGAAUA |
| siYB-1#3 | | CAGCCUAGAGAGGAUGGCAAUGAAG |
